# A cost/benefit analysis of self-care initiatives in the European Union: who gains, who loses?

**DOI:** 10.1186/2052-3211-8-S1-O5

**Published:** 2015-10-05

**Authors:** Anna-Theresa Renner, Julia Bobek, Herwig Ostermann, Peter Schneider, Sabine Vogler

**Affiliations:** 1 Department of Health Economics, Gesundheit Österreich GmbH (Austrian Public Health Institute), Vienna, 1010, Austria; 2 Department of Public Health, University for Health Sciences, Medical Informatics and Technology, Hall/Tyrol, 6060, Austria

## 

European health systems have increasingly come under pressure to implement cost-containment measures while simultaneously maintaining or even enhancing high-quality health care. In the field of medication of minor ailments, a promising approach to achieve this is initiatives promoting patient involvement (i.e. self-care). Therefore, the aim of the study was to analyse potential costs and benefits related to self-care oriented health systems in the European Union.

A cost/benefit analysis (CBA) was conducted covering modified access to prescription medicines, extended range of authorized prescribers and internet/telephone information portals. Costs and benefits were calculated for selected initiatives: minor ailment schemes (MAS), non-medical prescribing (NMP) and NHS Choices in England. The CBA covered four perspectives: patient, provider (physician, pharmacist), system and society. A standard costing approach was used to facilitate transferability of results.

In all studied initiatives, patients benefit from time savings due to avoided physician visits, compensating for occasionally higher out-of-pocket payments for medicines. Physicians are confronted with a negative benefit due to loss of income, which corresponds to a positive effect at the system level. If the initiatives' costs do not provide for additional remuneration for pharmacists, increased time for consultations will lead to a negative benefit for pharmacists.

In order to gain a positive societal net benefit, participation rates (in terms of patients with minor ailments refraining from a GP consultation due to an initiative) of 27.5% for MAS and 4.4% for NHS Choices are required. For NMP costs at providers' (i.e. pharmacists') levels are too high for a positive societal net benefit.

Self-care initiatives based on modified access schemes and information portals may lead to a societal benefit, whereas the mere extension of prescribing authority does not do so. As actual cost components of the initiatives (e.g. provider remuneration) and pharmaceutical reimbursement policies (e.g. prescriptions fees) are likely to affect savings and costs, these have to be considered when implementing a policy.

